# Religious and Medical Pluralism Among Traditional Healers in Johannesburg, South Africa

**DOI:** 10.1007/s10943-023-01795-7

**Published:** 2023-03-27

**Authors:** Michael Galvin, Lesley Chiwaye, Aneesa Moolla

**Affiliations:** 1 Health Economics and Epidemiology Research Office (HE2RO), Faculty of Health Sciences, University of the Witwatersrand, Johannesburg, South Africa; 2 Department of Psychiatry, Boston Medical Center (BMC), Boston, USA; 3 Harvard T.H. Chan School of Public Health, Boston, USA

**Keywords:** South Africa, Johannesburg, Traditional healers, Religious syncretism, Medical pluralism

## Abstract

Religion and spirituality are powerful social forces in contemporary South Africa. Traditional Health Practitioners (THPs) are commonly consulted for both spiritual and medical ailments as a first line of care. Many studies have assessed African traditional health seeking behaviors but few have examined beliefs, practices, and behaviors of THPs themselves. This study sought to explore spiritual worldviews among South African THPs. Semi-structured in-depth interviews were conducted with 18 THPs in Johannesburg, South Africa between January and May, 2022. Interviews were transcribed and translated into English. Data were managed using NVivo 12 software and thematically analyzed. The majority of THPs interviewed indicated that initiation as a THP was almost always preceded by a sickness accompanied by dreams/visions that represented an “ancestral calling” to become a healer. Most THPs also trained as both sangomas—who healed according to traditional beliefs—and prophets—who healed according to Christian beliefs. This reflects a syncretic relationship between traditional African beliefs and Christianity. However, not all churches are accepting of traditional beliefs and subsequently these THPs are members only at non-Pentecostal AIC churches who blend both African and Christian practices. Similar to these forms of religious pluralism melding Christianity and traditional beliefs, many THPs also often practice medical pluralism, mixing Western treatments with traditional practices/medicines. THPs are able to adapt elements of Western and African beliefs into healing practices that span multiple religious and medical fields. Thus, collaborative and decentralized healthcare services may be highly acceptable among such a pluralistic community.

## Introduction

Religion is a powerful social force in sub-Saharan Africa today (Bosire et al., 2021; Mzimkulu & Simbayi, 2006). With the arrival of Christianity since the late nineteenth century, African countries have been the site of religious syncretism and fusions of Christianity and African traditional practices and beliefs (Thornton, 2017). While it was the goal of early Christian missionaries to banish the “superstitions” of the local populations, local Christianity ultimately incorporated many of these practices, transforming into distinctive forms of organization and worship commonly referred to as African-initiated Churches (AICs) (Ashforth, 2005; Peltzer, 1999). In Africa today, these syncretic religious practices have given rise to pluralistic medical fields with newly emerging constellations of healing modalities that meld Western biomedical and traditional forms of diagnosis and treatment (Bosire et al., 2021; Hampshire & Owusu, 2013; Moshabela et al., 2016).

Medical pluralism refers to the concurrent usage of multiple treatment modalities and is common in places where biomedical and alternative treatments co-exist such as in sub-Saharan Africa (Sundararajan et al., 2020). In recent decades, this encounter between “traditional” and “biomedical” healing practices has attracted significant interest from researchers. South Africa in particular is a country that has elicited attention due to its pluralist system of health provision, with developed biomedical systems and relatively strong state support for traditional healers, referred to as Traditional Healing Practitioners (THPs) (Wreford, 2005b; Zuma et al., 2016).

THPs in South Africa work as faith healers in the Christian tradition—referred to as prophets (*amaprofeti*)—or as healers in the African traditions as diviners or herbalists—referred to as *sangomas* or *inyangas* (Kahn & Kelly, 2001; Wreford, 2005a). Sangoma is commonly used to designate a traditional healer in the African tradition, as many THPs utilize both herbs and divination, and will therefore be the term used in this text (Thornton, 2017). With regards to illness, THPs generally treat conditions brought about by causes such as spirits, ancestors, sorcery, and bewitchment (Ashforth, 2005; Audet et al., 2017). In addition to curing illness, THPs also assist clients in making decisions, finding lost or stolen objects, resolving problems, attracting or retaining lovers, getting a job, or for protection (Thornton, 2017).

Previous studies have estimated that between 70 and 80% of South Africans consult THPs for the treatment of illness (Berg, 2003; Crawford & Lipsedge, 2004; Kahn & Kelly, 2001; Mzimkulu & Simbayi, 2006; Robertson, 2006; Thornton, 2009). Additionally, the vast majority of South Africans who consult THPs are of Black African ethnicity. While many studies have assessed health seeking behaviors of the South African population within this pluralistic religious and medical environment, few have examined pluralism with regards to the beliefs, practices, and behaviors of THPs themselves (Hampshire & Owusu, 2013; McMillen, 2004). This study seeks to examine pluralistic religious and medical practices among THPs so as to understand how spiritual worldviews among traditional healers have been revitalized to meet contemporary challenges of health and well-being in South Africa.

## Methods

### Study Setting and Design

This study enrolled 18 THPs practicing in townships surrounding Johannesburg, South Africa between January and May 2022. In particular, the THPs interviewed lived and worked in either Soweto, Katlehong, or Vosloorus townships. A qualitative study design was utilized, with semi-structured interviews to elucidate THP understandings of illness and healing within pluralistic religious and medical contexts. An interview guide was developed with guidance from two THPs associated with the Health Economics and Epidemiology Research Office (HE^2^RO) at the University of the Witwatersrand. This guide was pilot tested on two THPs in January 2022; however, these responses are not included in this analysis.

### Recruitment and Sampling

During the recruitment process, participants were informed that this study was associated with a local university in Johannesburg. Participants were recruited using a snowball sampling technique in which THPs were referred via word of mouth. Participating healers were asked if they could provide contact information for other healers working in Johannesburg, as the sector is largely informal. This sampling strategy allowed researchers to maximize representation of healers from throughout the townships surrounding Johannesburg. A sample size of 18 was guided by the concept of data saturation in which interviews no longer reveal new content and participants often repeat information previous interviewees reported. Transcripts of interviews were analyzed on an on-going basis throughout the study process to ensure that emerging themes could be understood and explored further.

### Data Collection

Eligibility criteria included (a) being 18 years of age or older; (b) able to provide informed consent and consenting to recording of the interview; and (c) recognized as an official THP after completing *thwasa* (traditional healer training). Interviews lasted between 35 and 60 min and were conducted by the study PI and study research assistants – who are also practicing THPs or in training – at the home or workplace (*indumba*) of the healer. Research assistants were fluent in English, isiZulu, and isiXhosa and could ensure real time translations in the event the participant did not speak fluent English.

### Data Analysis and Ethical Considerations

Interviews were consequently transcribed and translated into English. Two authors reviewed all English transcripts for content relevant to religious and medical pluralism in THP practices. This analysis utilized a directed content analysis method that combines deductive and inductive aspects of codebook development (Hsieh & Shannon, 2005). Themes were developed and refined resulting in the generation of a final set of codes. Both researchers subsequently utilized NVivo 12.0 to code the transcripts. Inter-coder reliability was used to ensure common understandings of concepts and codes.

This study received approval from the Human Research Ethics Committee at the University of the Witwatersrand (#M210815) and the Research Committee of Johannesburg Health District (#GP_202111_059). Participants provided written informed consent and were compensated 100 rand (roughly $7) for their time. This amount was determined together with the University of the Witwatersrand and HE^2^RO. All COVID-19 protocols were followed during the collection of study data.

## Results

Eighteen THPs were interviewed for this study, including 9 males and 9 females. Healers ranged between 21 and 74 years old with an average age of 39 years old. THPs also had between 2 and 48 years of experience as a healer with an average of 16 years. See Table 1 for Participant Demographic Characteristics.

**Table 1:** Participant demographic characteristics

Participant number	Gender	Age	Years working as a healer	Type of healer	Location
1	Male	40	27	Apostolic prophet and sangoma	Soweto
2	Male	30	16	Apostolic prophet and sangoma	Katlehong
3	Male	39	18	Apostolic prophet and sangoma	Katlehong
4	Male	29	14	Prophet and sangoma	Soweto
5	Male	41	11	Prophet and sangoma	Soweto
6	Male	26	12	ZCC prophet and sangoma	Vosloorus
7	Male	21	7	Zion prophet and sangoma	Soweto
8	Male	28	10	Apostolic prophet and sangoma	Katlehong
9	Male	30	2	Apostolic prophet and sangoma	Soweto
10	Female	38	16	Apostolic prophet and sangoma	Soweto
11	Female	74	48	Shembe prophet and sangoma	Vosloorus
12	Female	62	13	Zion prophet and sangoma	Soweto
13	Female	37	12	Apostolic prophet and sangoma	Soweto
14	Female	50	25	Sangoma	Soweto
15	Female	68	38	Sangoma	Vosloorus
16	Female	29	10	Apostolic prophet and sangoma	Soweto
17	Female	38	5	Apostolic prophet and sangoma	Soweto
18	Female	28	3	Sangoma	Soweto

### THPs as Sangomas and Prophets

Fifteen of the 18 THPs we interviewed were both sangomas and prophets, reflecting a syncretic relationship between traditional African beliefs and Christianity. For the difference between sangomas and prophets refer to Table 2. Of the 15 sangomas that also worked as prophets, 9 were with the Apostolic church, 3 with the Zion Christian Church (ZCC), 1 with Shembe, and 2 with other independent churches. THPs who were both sangomas and prophets described having to ensure a balance between the two roles, often referred to as “gifts”:

We have to balance the two, if you have two gifts. If you have two kids and you give more attention to one than the other, one will feel left out. That’s not how it’s supposed to be, you have to balance them out. Make them feel equal. That’s how it works with the traditional part of it and the church part of it. (Participant 8)

**Table 2:** Differences between Sangomas and prophets

Sangomas	Prophets
Practice healing primarily according to African traditional beliefs	Practice healing primarily according to Christian or Biblical beliefs
Receive a calling through illness or dreams with people in traditional dress, drums, or ancestral animals (lions, snakes)	Receive a calling through illness or dreams with candles, being in church, holding a Bible, or placing hands on people to heal them
Inherited from ancestor who was a sangoma	Inherited from ancestor who was a prophet
Are initiated as healers through *thwasa* training	Are initiated as healers through churches
Primarily heal with *muthi* (herbs)	Primarily heal with holy water and anointing oil

Another THP referred to switching between the two roles as being “like acting”:

For example, last weekend I had a ceremony where I was appointed as archbishop of a church. And then this week I have a ceremony with traditional healers, it’s another world. So when I work in the church I just put my mind on that thing. It’s like acting. Like a character on a stage. This week, episode 2, he or she is a pastor. Episode 3, etc. It’s just like that. You adjust to that environment in that present moment. (Participant 1)

There was no designated order in which a THP becomes a sangoma or a prophet as 6 became sangomas first, 6 became prophets first, and 3 were initiated into both at the same time. Usually, the THP would become initiated into one of the two healing traditions first based on their family or community. As one recounts:

My grandmother was a church person. I played drums at the church. And then all of a sudden I would see things, I had visions. I said to the pastor, ‘when you were talking about this and this, I was seeing this and this.’ The congregation also was amazed. That’s how it came about. I graduated in the prophecy. (Participant 8)

Others described initiating into a second healing tradition after repeated sickness that wasn’t cured by the first initiation, or having specific dreams or visions associated with either sangoma or prophetic healing.

### Sickness and Dreams as Calling to Become a Healer

Initiation as a THP is almost always preceded by a sickness that represents an “ancestral calling” to become a healer. Sixteen of the 18 healers interviewed reported experiencing their calling via illness. Most healers explained that the illness related to their ancestral calling was one that “doctors cannot treat.” Once it was established that doctors could not treat the illness, the individual would go to a sangoma who would confirm that it was a calling:

The family didn’t understand. We went to the doctors and nothing was helping. Then we went to see a traditional healer and discovered it was a calling. I couldn’t walk, I was weak. The doctors said there was nothing they could see on their end. And that’s when the healer said I have the calling. (Participant 13)

As mental illness is often more difficult for medical professionals to diagnose and treat, many healers experienced mental illnesses which they interpreted as their ancestral callings:

They come as a mentally disturbed person. People say, oh this person is mad. But you have to check what’s wrong, why they are like this. Some want to kill themselves, some want to be alone all the time. Some leave their marriages. Everything goes back to how the calling comes through somebody’s body. You have to be very careful to see why someone is ‘mad’ in relation to their ancestors. (Participant 14)

Besides mental illness, other THPs reported sicknesses such as seizures, fainting spells, back pain, headaches, chronic fatigue, and vision problems as their callings. The sickness caused by the ancestral calling is not considered the same as a normal illness. Rather it is a manifestation of the ancestors (*amadlozi*) compelling the individual to follow in the footsteps of one of their relatives who was a THP in the past:

I didn’t know I had a calling originally to be a traditionally healer or sangoma. But I was sick. They thought I was mentally disturbed. I was doing funny things, strange things. Then my grandmother took me to a traditional healer in Eastern Cape province in 1994, I was 11 years old. This traditional healer said I had a calling. I didn’t know there was anyone in my family who was a traditional healer except for my aunt. She was a traditional healer. So I took this from my aunt, she passed away in 1986. (Participant 1)

All THPs reported that upon starting traditional healing initiation school (*thwasa*) the illness immediately disappeared. As one describes:

Because of the back pain, I went to several healers. Spiritual, traditional, and faith. I was told that I have a calling that I must heed, and do traditional healing initiation. The pain stopped when I started receiving treatment which was aimed at becoming a traditional healer. (Participant 11)

*Thwasa* is thus seen as the only cure for supernatural forms of illness that indicate the individual has a calling.

In addition to sickness, THPs usually also have dreams or visions which indicate an ancestral calling. There is a distinction between dreams which indicate a calling to become a sangoma and a calling to become a prophet however. As one describes:

In prophecy you dream about water, candles, singing in church, yourself flying, that represents you being a prophet. But being a sangoma you dream about ancestral animals like lions, snakes, being on the water. (Participant 9)

Other common dreams reported which indicate a calling include being top of a mountain or in a river, placing your hands on people to heal them, being under water, seeing people in sangoma dress or hearing drums (for calling as a sangoma) and holding a Bible (for calling as a prophet). In addition to dreams, some THPs reported visions in which they could foresee the future. As one described:

If there was going to be a death in the family, I would dream about it and tell them and it would happen. The day my father passed on I had a dream his spirit was coming out of his body, I woke up and told my mom my dad is no more. And thirty minutes later they called us to alert us that he was no more. (Participant 5)

While nearly all THPs have a sickness which indicates their calling, many also have dreams; however, few reported prophetic visions in which they could predict future events.

### THPs, Tradition, and Churches

THPs who practice as both sangomas and prophets are members of non-Pentecostal AIC churches. Sometimes these churches are referred to locally as *omoya* (*amaChurch omoya*)—or “spirit” in isiZulu—which can be differentiated from Pentecostal or “born again” (*bazalwane*) churches. For the differences between AIC churches refer to Table 3. Unlike Pentecostal churches, these churches generally welcome sangomas. Conversely, Pentecostal churches largely do not allow sangomas as they often view them as evil. As one described:

[If our church was *bazalwane*] we wouldn’t be able to pray to the ancestors and burn *impepho* [traditional incense]. I wouldn’t be able to go there if it was *bazalwane*. They don’t like sangomas. (Participant 7)

**Table 3:** Differences between types of AIC churches

Non-Pentecostal	Pentecostal
Not considered charismatic, or “born again” churches	Considered charismatic, or “born again” churches
Allows sangomas to attend and practice within the church	Does not allow sangomas to integrate the church
Church prophets also commonly practice as sangomas	Church prophets do not practice as sangomas
Incorporates traditional African beliefs such as ancestor worship within Christian practices	Does not allow ancestor worship and views many traditional African beliefs and practices as “evil” or “demonic”

Two of the THPs interviewed said they had to leave their Pentecostal churches when they decided to become sangomas as they were no longer welcome. Another described having to move out of his mother’s house—as her side of the family was Pentecostal—when he realized he had a calling and decided to become a sangoma. He consequently relocated to his father’s house whose side of the family was more involved with traditional religious practices. He recounts:

I went home to my mother and told her [I had a calling] and she said they didn’t want to interfere. She said ‘we don’t have that here’ [sangomas] since her family is *bazalwane* [Pentecostal]. She said if you want those things go to your dad’s house. (Participant 5)

In light of this rejection by Pentecostal Christians, many sangomas described feeling resentment toward “born again” churches and saw them as turning away from their roots:

These people run away from African culture. They think being born again or *bazalwane* [Pentecostal] they can push these things away. They try to brush these things off. But these things are alive and are living with us. They are our main purpose of being on earth. Connecting with our ancestors (*amadlozi*). And they don’t want that. (Participant 4)

Non-Pentecostal AIC churches on the other hand not only welcome sangomas but often blend traditional African practices with Christian practices. One described how at his church they also burn traditional incense and pray to their ancestors during church services. Similarly, as healers, THPs describe using both the Christian God and traditional practices together when performing rituals. As one described:

At the end of the day we are all God’s creation. Jesus, God. So God comes first. Whatever we do, we throw the bones [a sangoma divination practice], we pray. With no guidance from God we are nothing. (Participant 4)

### Traditional Beliefs and Western Medicine

Similar to these forms of religious pluralism melding Christianity and traditional beliefs, many THPs also often practiced medical pluralism, mixing Western treatments with traditional practices. One THP was both a sangoma, a prophet, and a practicing nurse and described how she often encourages patients from the hospital to come visit her for traditional healing services after hours:

I’m a sangoma and a professional nurse. As a nurse we use Western medicine. With sangomas we take it straight from the roots, it’s natural, there’s no manufacturing… I don’t see any conflict. I get a patient at the clinic and then I tell him or her to come see me later [as a sangoma]. If they want to come see me later. So I send them to the clinic where the medicine is [dispensed]… And get the medication and they come back to me. We deal with it as a two way street [traditional and Western treatments simultaneously]. (Participant 17)

This reflects a commonly held belief among THPs that some illnesses are supernatural and for traditional healers to treat, and other illnesses are natural and for doctors to treat. By encouraging patients to go through both treatments, they are ensuring a cure regardless of the illness’ etiology. Even if a patient is diagnosed with a commonly known illness such as HIV or Tuberculosis at the hospital, THPs argue that these could actually be supernatural illnesses disguised as Western ones, thus requiring traditional intervention:

The *muthi* [herbs] curses can cause you to have cancer. It can cause you to have HIV. The spell that’s cast on you is so heavy, any bad thing can happen to you. All the negative energy and misfortunes. So if you go to Western medicine to get treatment and it’s not working, you will come back to me and I can tell you if someone cast a spell on you. The [doctor’s] medication will not work. But we fight with that curse. (Participant 4)

Several THPs argued that they had the power to cure illnesses such as HIV as it was, at times, of supernatural origin. In the case of these supernatural illnesses, some THPs argue that a doctor’s treatments could not only be ineffective but could also be harmful:

So doctors are Western, and sangomas are traditional right? There are sicknesses that a doctor cannot see. For example, if you are being troubled by an ancestor. Like if you have a calling and need to *thwasa*. You will be getting sick and having headaches or lots of flu or asthma, sometimes it’s ancestors and not really asthma. But the Western doctor will think it’s asthma but it’s really *amadlozi* [ancestors]… If they [doctors] think it’s asthma they’ll give you an asthma pump and it will just make you worse. (Participant 2)

In spite of these differences, almost all THPs interviewed viewed collaboration with Western medicine positively. While they saw many similarities between the two professions, they also understood that doctors go through many years of rigorous training and that THPs complete significantly less years of training:

The only difference is doctors are qualified and sangomas only need to go for initiation. We don’t go to university for 6 years, we just go to initiation [*thwasa*] for 6 months or 10 months and we’re done. (Participant 18)

In addition to *thwasa* however, many THPs had completed short medical training courses at local hospitals on topics ranging from HIV/AIDS, COVID-19, cancer, first aid, fundamental nursing, STIs, TB, hygiene, nutrition, family planning, and basic counseling. These courses were largely organized by local THP associations of which many healers are members. Many healers reported desiring more training in biomedical approaches. Almost all healers also encouraged patients to get tested for HIV at a clinic or hospital prior to starting treatment with them due to the high community infection rates in South Africa. This reflected an almost universal willingness to ensure that their patients are engaging with doctors simultaneously to receiving their treatment as traditional healers.

## Discussion

### THPs as Sangomas and Prophets

The THPs interviewed in this study represent a common trend in contemporary Southern Africa in which many sangomas practice faith healing as prophets as a form of “African syncretic Christianity” (Thornton, 2009, 2017, p. 5). This movement started with the rise of early twentieth century African-initiated Churches (AICs) which broke away from more Western-oriented churches brought by European missionaries and share a common theory of health and disease with traditional African beliefs (Mzimkulu & Simbayi, 2006). As a result, a large segment of South African religious practices today are an “amalgamation of traditional cosmology and Christianity” (Peltzer, 1999; Kahn & Kelly, 2001, 38). Others have described them as “syncretic cults… mixing half-baked Christian teachings with indigenous religious practices to create a new hybrid form of syncretic spirituality” (Ashforth, 2005, p. 189). In this sense, the sangoma/prophets we interviewed are seen as persons with clairvoyant healing powers operating within “African church cosmology” (Kahn & Kelly, 2001; Sorsdahl et al., 2009). This reflects broader trends within African societies in which virtually no religious practices and beliefs are entirely “pure,” or free from African traditional ideas that pre-existed the arrival of Christianity; rather, this syncretism is part of a “broader cultural fusion” that represents a vibrant market for religious ideas in which few are effectively “mono-religious” and almost everyone is continually “shopping” within a religiously pluralistic environment (Atindanbila & Thompson, 2011; Thornton, 2017, p. 157).

### Sickness and Dreams Represent a Calling

The sangoma/prophets we interviewed were almost all called to their profession via illness. This calling means that the individual’s ancestors are compelling him or her to become a healer in order to use supernatural powers to explain misfortune and illness and offer guidance on how to appease ancestors (Kahn & Kelly, 2001; Thornton, 2017). Whether as sangomas or prophets, THPs often use their powers to treat “witchcraft/sorcery related problems,” highlighting the influence of traditional beliefs on African syncretic Christianity as well (Peltzer, 2001, p. 3). For sangomas in particular, the calling in the form of an illness derives from the worldview of pre-colonial African belief systems in which the ancestral calling is handed down from generation to generation (Crawford & Lipsedge, 2004; van der Zeijst et al., 2021a). By completing the *thwasa* training, one is answering the ancestral call and continuing the familial tradition of healing work. As prophets also often receive a calling via illness, we can see the influence traditional African beliefs have had on the evolution of local Christianity.

As described by the THPs interviewed above, the calling is most often related to mental illness with psychotic disorders the most prominent manifestation, and mood disorders and anxiety disorders to a lesser extent (Sorsdahl et al., 2010a; van der Zeijst et al., 2021a). Mental illnesses are more difficult for medical professionals to diagnose and treat as symptoms are determined by self-report rather than blood tests, x-rays, or other biomarkers. For this reason, individuals experiencing symptoms of mental illness such as social withdrawal, strange behavior, or unexplained aggression are often told they have a calling and should undergo *thwasa* (Atindanbila & Thompson, 2011). Lastly, many THPs also described their calling as coming in the form of dreams. From a cultural perspective, dreams are regarded as “messages from the ancestors” and represent an essential connection between conscious and unconscious life (Berg, 2003, p. 201). Similar to dreams, visions and unseen voices can also be interpreted as ancestors or other spirits seeking to communicate with the living (Ashforth, 2005). Dreams such as those, including scenes from the Bible or ancestral animals, indicate a calling specific to the prophecy or rites of sangomas.

### Pentecostal (*bazalwane*) and Non-Pentecostal AIC Churches

While it is difficult to measure, there are estimated to be hundreds of thousands of “prophets” in South Africa (Ashforth, 2005). Many of these prophets are part of “born again” (*bazalwane*) or Pentecostal churches which represent the fastest growing sector of South African Christianity (Ashforth, 2005). The largest of these Pentecostal churches is the Zion Christian Church (ZCC) designated by a yellow star that members wear; this differentiates it from other Zion or ZCC churches which are not “born again” highlighting complex denominational divisions in countries such as South Africa (Rio et al., 2017; Thornton, 2017). In other words, many churches share similar names or titles but have different approaches to incorporating sangomas and other traditional African beliefs such as ancestor worship.

There were no Pentecostal prophets interviewed for the purposes of this study, as *bazalwane* church members do not generally associate themselves with sangomas who they “revile as ‘primitive’, dirty and spiritually dangerous” (Thornton, 2009). In fact, much of the work of Pentecostal prophets is directed toward counteracting the work of witches, sorcerers, and other figures associated with traditional African belief systems (Ashforth, 2005, p. 187). This is a form of evangelism that some experts on African Pentecostalism have termed “confrontationist,” in that they view local African beliefs through the prism of Christian demonology (Rio et al., 2017, p. 3). *Bazalwane* beliefs are still arguably syncretic mixes of Christianity and traditional beliefs however, as “in their attempt to combat witchcraft, they were instead drawn into the witchcraft world” (Rio et al., 2017, p. 12). In other words, in demonizing traditional beliefs they are acknowledging them as active forces within their own religious worldview.

Similarly, with regards to ancestor veneration, *bazalwane* believe “people who venerate their ancestors… do so because they are kept in darkness by the devil and do not realize the vastly superior power available to them should they become saved [by Jesus]” (Ashforth, 2005, p. 209). While the prophets we interviewed for this study also perform similar roles in combating witchcraft, they do not view traditional African beliefs in a negative light. Rather, they incorporate traditional African rituals and customs into their work as Christian prophets and see them as another tool in the spiritual toolbox (Thornton, 2017; van der Zeijst et al., 2021a). In this sense, the prophets we interviewed simultaneously pray to the Christian God along with their ancestors when seeking spiritual guidance (Ashforth, 2005). Some researchers argue that with the rise of Pentecostal and “born again” churches in sub-Saharan Africa, THPs are increasingly positioning themselves to appeal to the *bazalwane* anti-traditional rhetoric (Hampshire & Owusu, 2013).

### Medical Pluralism and Collaboration

Similar to religious pluralism with the South African spiritual landscape, medical pluralism is also common among both the general population and THPs. Although Western biomedicine has become increasingly accepted in recent decades, belief in supernatural explanations of illness and misfortune remain widespread (Sundararajan et al., 2021a, 2021b; van der Zeijst et al., 2021b). This distinction is particularly strong with regards to the foundational distinction between “natural” illnesses and “supernatural,” “man-made,” or “African” illnesses (*ukufa wa Bantu* or *imisebenzi yabantu*) (Ashforth, 2005). While natural illnesses are susceptible to treatment by Western medicine, supernatural illnesses are thought to only respond to the intervention of healers deploying spiritual powers. In this sense, even THPs hold multiple explanatory models when it comes to illness as they refer patients they determine are afflicted with “natural” illness to doctors for treatment (Sorsdahl et al., 2010a). Thus, as we saw with the THP who also worked as a professional nurse, the possibility of simultaneously subscribing to and participating in disparate healthcare systems is commonly accepted.

In another study examining medical pluralism among psychiatric nurses in South Africa, researchers found that the nurses subscribed to pluralistic systems which drew on both African and Western cultural worlds without an absolute allegiance to either (Kahn & Kelly, 2001). This reflects the view that different practitioners can fulfill different needs. An additional distinction may be the role THPs play in African societies in which they not only work to assuage the symptoms of the illness but also provide an “explanation” for the illness (Wreford, 2005a). Some experts argue that this may justify the continued popularity of THPs even in places where biomedical treatment is relatively easy to access (Crawford & Lipsedge, 2004; Ndetei et al., 2013).

Due to the strong tendencies toward medical pluralism already in place among THPs in this region, collaboration between doctors and THPs is overwhelmingly supported by the latter, as evidenced by the results of this study. A significant literature already exists exploring this subject in South Africa with many researchers in support of such arrangements (Campbell-Hall et al., 2010; Moshabela et al., 2016; Schierenbeck et al., 2018; Sorsdahl et al., 2010a; van der Zeijst et al., 2021b; Veling et al., 2019). This reflects the notion that traditional healers are extensively patronized, have a large clientele, maintain significant respect within local communities, and therefore should be viewed as “bottle openers” rather than “bottlenecks” for collaboration and treatment (Sundararajan et al., 2021a). Recent research has found that collaborations have been successful between biomedical healthcare workers and traditional healers throughout sub-Saharan Africa (Audet et al., 2013; Baheretibeb et al., 2021; Gureje et al., 2020; Musyimi et al., 2017; Pascoe et al., 2013; Sundararajan et al., 2021b; Veling et al., 2019).

Nevertheless, it is important to point out that there are also many researchers who warn against potential pitfalls of collaboration between biomedical and traditional health practitioners. The lack of a uniform code of ethics or training among THPs to protect patients, reports of exorbitant fees charged to patients, physical or sexual abuse at the hands of THPs, reports of acute poisoning caused by traditional herbal remedies, delays in receiving evidence-based treatments, and lack of trust between traditional healers and biomedical workers are just some of the issues that experts warn could plague potential collaboration (Akol et al., 2018; Atindanbila & Thompson, 2011; Kajawu et al., 2016; Louw & Duvenhage, 2016; Ojagbemi & Gureje, 2021; Robertson, 2006; Sorsdahl et al., 2010b; Stewart et al., 1998).

In spite of these challenges, there remains widespread interest in exploring medically pluralistic models of treatment in contemporary South Africa (Kahn & Kelly, 2001). This can potentially be achieved through the opening of dialogues and conducting workshops with both THPs and biomedical healthcare workers in presence, as well as suggestions of both parties working together to discuss points of synergy where their work could dovetail within the different levels of care. This could also include biomedical healthcare workers building enough rapport with their own patients to be able to ask them in a more open-ended manner if they are actively seeking out or are already taking alternative modes of treatment, and then accommodating this into treatment planning.

## Limitations

As this study is qualitative and only interviewed 18 THPs, it is not meant to be generalizable to the general population of healers in Johannesburg or Southern Africa. Rather, this study provides specific and detailed information about a unique population of interest. For example, this study did not include *bazalwane* prophets, who also work as spiritual healers in this context, and is therefore not generalizable to them. While there are many similarities with other studies that have examined the beliefs and practices of healers in Johannesburg and Southern Africa, more research is needed to fully assess the constantly evolving relationship between religious and medical pluralism among these populations in order to understand how they impact trends in spirituality and health seeking behaviors in the region.

## Conclusion

Contemporary South Africa is a site of increasingly complex religious and medical pluralism, giving rise to newly emerging constellations of beliefs systems and therapeutic possibilities. The THPs interviewed for this study adapt elements of Western and African beliefs and healing practices in eclectic ways to span multiple religious and medical fields, self-positioning across a range of traditions and forms of knowledge. Recent research highlights that collaborative and decentralized healthcare services would be highly acceptable among pluralistic communities (Sundararajan et al., 2020). While few studies have tested collaborative healthcare interventions between THPs and biomedical practitioners, this study shows that there are multiple avenues for cooperation with this new generation of THPs in South Africa.
